# Surgical stabilisation of rib fractures in non-ventilated patients: a retrospective propensity-matched analysis using the data from the trauma registry of the German Trauma Society (TraumaRegister DGU^Ⓡ^)

**DOI:** 10.1007/s00068-024-02756-9

**Published:** 2025-01-24

**Authors:** Michael D. Huelskamp, Helena Duesing, Rolf Lefering, Michael J. Raschke, Steffen Rosslenbroich, TraumaRegister DGU

**Affiliations:** 1 Department for Trauma-, Hand- and Reconstructive Surgery, University Hospital Münster, Münster, Germany; 2 Institute for Research in Operative Medicine (IFOM), University Witten/Herdecke, Cologne, Germany; 3 Committee on Emergency Medicine, Intensive Care and Trauma Management (Sektion NIS) of the German Trauma Society (DGU), Berlin, Germany

**Keywords:** Surgical stabilisation of rib fractures, SSRF, Flail chest, Rib fractures, Non-ventilated

## Abstract

**Purpose:**

Severe thorax trauma including multiple rib fractures and flail chest deformity are leading causes of death in trauma patients. Increasing evidence supports the use of surgical stabilisation of rib fractures (SSRF) in these patients. However, there is currently a paucity of evidence for its use in non-ventilator-dependent patients.

**Methods:**

A retrospective propensity-matched analysis of the data of the TraumaRegister DGU^®^ for non-ventilator-dependent patients with severe rib injury (abbreviated injury score ≥ 3) was performed. Subgroup analyses with respect to injury severity score, American society of anaesthesiologists physical status classification and age were performed. Furthermore, the effect of time to surgery was analysed.

**Registration:**

TR-DGU project ID 2023-007; ClinicalTrials.gov protocol ID: NCT06464289.

**Results:**

SSRF led to reduced mortality compared to conservative treatment (1.6% vs. 4.8%; *p* = 0.002) and in comparison to the mortality prognosis of the revised injury severity classification II (RISC II) of 5.2%. Interestingly, SSRF was associated with increased length of hospital and intensive care unit stay, higher rates of organ failure and secondary intubation. The patients with organ failure received SSRF later than those without organ failure.

**Conclusion:**

Here we report on the largest currently published dataset of non-intubated patients receiving SSRF, which showed reduced mortality in the SSRF cohort. The data indicates that SSRF is a viable treatment option for non-intubated patients. The observed late surgical time points, which may be due to cross over after failed conservative treatment, might be the cause for the observed increased rate of organ failure.

## Introduction

Trauma is the most common cause of death in patients under 45 years of age [1, 2]. Thoracic trauma is the third most common primary and most common secondary cause of death [3–5]. A recent study found that 6% of trauma patients presenting to the emergency room had at least one fractured rib. The incidence rate of patients requiring hospital admission due to rib fractures was 26 per 100,000 patient-years [6].

A variety of different mechanisms contribute to the morbidity and mortality caused by rib fractures. These include pain-induced hypoventilation, pneumothorax, which occurs in approximately 1/3 of rib fracture patients, haemothorax as well as pulmonary contusions [7]. Furthermore, in patients with severe thoracic trauma, especially in flail chest (3 or more contiguous ribs fractured in 2 or more places) the breathing biomechanics may be affected even under adequate analgesia [8].

Historically, rib fractures were treated conservatively including systemic analgesia, epidural catheterisation and intercostal nerve blocks and if necessary by positive pressure ventilation to achieve “internal pneumatic stabilisation” [8]. More recently, the concept of surgical stabilisation of rib fractures (SSRF) has become a topic of frequent discussion and extensive investigation [9].

Within the available literature there appears to be a consensus that patients with flail chest and those patients that are dependent on mechanical ventilation due rib fractures may benefit from SSRF [9–11]. There is, however, currently a paucity of evidence on the application of SSRF in patients that do not fall into one of these two groups [10, 11].

Physiological considerations lead to the conclusion that SSRF may be suitable to reduce pain and to improve both baseline and maximal respiratory function in patients with rib fractures. This may lead to lower rates of pulmonary complications and to improved physiological functioning in the short and long term. Only a few recent studies have specifically investigated SSRF in non-intubated patients [12–14]. These found some evidence that the indications for SSRF may be expanded to non-ventilator-dependent patients, although their findings were not entirely consistent. One study found a reduction in pain [12], one found early earlier return to work [13] and one study found improved respiratory function after SSRF [14].

The aim of this investigation, therefore, was to analyse the data from the trauma registry of the German Trauma Society (TraumaRegister DGU^®^; TR-DGU) with respect to epidemiology and outcomes of the group of patients with severe rib fractures (rib fracture abbreviated injury score (AIS) ≥ 3) that were not intubated pre-clinically or in the emergency department. We hypothesised that SSRF could lead to reduced mortality, reduced intensive care unit length of stay (ILOS) and where secondary intubation was needed reduced duration of mechanical ventilation in these patients.

## Methods

### TraumaRegister DGU® (TR-DGU)

The TraumaRegister DGU^®^ of the German Trauma Society (Deutsche Gesellschaft für Unfallchirurgie, DGU) was founded in 1993. The aim of this multi-centre database is a pseudonymised and standardised documentation of severely injured patients.

Data are collected prospectively in four consecutive time phases from the site of the accident until discharge from hospital: (A) Pre-hospital phase, (B) Emergency room and initial surgery, (C) Intensive care unit and (D) Discharge. The documentation includes detailed information on demographics, injury pattern, comorbidities, pre- and in-hospital management, course on intensive care unit, relevant laboratory findings including data on transfusion and outcome of each individual. The inclusion criterion is admission to hospital via emergency room with subsequent intensive or intermediate care unit (ICU/ICM) care or admission to the hospital with vital signs and death before admission to ICU.

The infrastructure for documentation, data management, and data analysis is provided by AUC - Academy for Trauma Surgery (AUC - Akademie der Unfallchirurgie GmbH), a company affiliated to the German Trauma Society. The scientific leadership is provided by the Committee on Emergency Medicine, Intensive Care and Trauma Management (Sektion NIS) of the German Trauma Society. The participating hospitals submit their data pseudonymised into a central database via a web-based application. Scientific data analysis is approved according to a peer review procedure laid down in the publication guideline of TraumaRegister DGU^®^.

The participating hospitals are primarily located in Germany (90%), but a rising number of hospitals of other countries contribute data as well (at the moment from Austria, Belgium, China, Finland, Luxembourg, Slovenia, Switzerland, The Netherlands, and the United Arab Emirates). Currently, more than 38,000 cases from almost 700 hospitals are entered into the database per year. Participation in TraumaRegister DGU^®^ is voluntary. For hospitals associated with TraumaNetzwerk DGU^®^, however, the entry of at least a basic data set is obligatory for reasons of quality assurance.

### Study design and inclusion criteria

This study follows a retrospective propensity-matched design based on the data of the TraumaRegister DGU^®^ (TR-DGU). The abbreviated injury score (AIS), according to the manual “Abbreviated Injury Score© 2005 Update 2008”, was used to classify injury severity [15]. Patients with severe rib injuries (AIS ≥ 3), not intubated before admittance or in the emergency department (ED) between 2013 and 2022 were included. The detailed inclusion criteria and intervention group definitions are given in the PICO statement:

### PICO statement

- **Patients**: primary admission with available standard data set of the TR-DGU, rib fracture AIS ≥ 3, no intubation pre-clinically or in ED. Patients that deceased within 1 week under palliative treatment regime were excluded.
- **Interventions**: SSRF by any technique (including but not limited to plate, thoracoscopic, intramedullary osteosynthesis).
- **Control**: Any non-operative treatment (including but not limited to analgesia, physiotherapy, treatment in an intensive care unit (ICU).
- **Outcomes**: Primary: Mortality, ICU length of stay (ILOS) and duration of mechanical ventilation. Secondary: Rate of secondary intubation, hospital length of stay (HLOS), rate of organ failure (OF) (lung OF, single OF, multiple OF (MOF)) and sepsis, outcome at discharge according to the Glasgow outcome scale (GOS).

### Patients

The dataset of the TR-DGU between 2013 and 2022 was used. Only patient records with the standard data set were included, since the reduced basic data set does not contain the needed information with respect to surgical treatment. Patients were identified based on the presence of an AIS score for a serial rib fracture, defined as three or more contiguous fractured ribs (450203.3), unilateral small flail chest thorax, defined as three or more contiguous segmental rib fractures on one side (450211.3), unilateral large flail chest, defined as more than five contiguous segmental rib fractures on one side (450213.4) and bilateral flail chest, defined as segmental rib fractures bilaterally (450214.5). Patients that were intubated prior to arrival to the hospital or in the ED were excluded (identified by the “endotracheal intubation” and “surgical airway” data fields in section A for the prehospital and section B for the ED phases of treatment). Furthermore, patients that deceased within one week of hospital admission under a palliative/non-curative treatment regime (identified by the corresponding variable in the outcome section D of the patient’s dataset) were excluded, in order to limit confounding by inclusion of patients with reduced treatment intensity into the control cohort. No restriction was placed on patient age.

The patients were assigned to the surgical group via an automated search based on the presence of a surgical procedure code indicating osteosynthesis and/or by an appropriate free text description of the procedure, linked to the rib fracture diagnosis. The following types of procedures from procedure group 7 of the TR-DGU dataset were considered to indicate osteosynthesis: cerclage/tension-band/wire osteosynthesis; external stabilisation; intramedullary stabilisation; nail osteosynthesis; plate osteosynthesis; screw osteosynthesis. All patients not included in the surgical group were included in the control group.

### Statistical analysis

Patients were divided in two groups, the SSRF and the non-operative group, as described above. A logistic regression model with SSRF as dependent variable was used to estimate the propensity score. The rounded score was used to perform 1:3 propensity matching. The following variables were used:

- Rib AIS 3 / 4 / 5.
- Age ≥ 60 years.
- Head AIS ≥ 3.
- Pneumothorax.
- Haemothorax.
- Lung laceration.
- Scapula fracture.
- Chest tube in ER.
- Trauma centre level 1.

The propensity score describes the probability of receiving SSRF, expressed as a percentage. The matching was performed as an exact match based on the rounded score.

For descriptive statistics mean with SD and median with first and third quartiles were used for continuous measurements, and n with percent was used for categorical variables. The chi-squared and Mann-Whitney U-tests were used as appropriate and *p* < 0.05 was considered statistically significant. Where data points relevant to a specific analysis were missing, the patient dataset was excluded for this specific analysis. Exploratory subgroup analysis of influence factors identified from literature was performed. Due to the exploratory nature of these analyses and in order to avoid multiplicity of testing no p-values were reported in these cases. All statistical analyses were performed with SPSS^®^ (Version 24, IBM Inc., Armonk, NY, United States).

### Reporting and registration

This study was performed and reported in accordance with the publication guidelines of the TraumaRegister DGU^®^ as well as according to STROBE guidelines for case-control studies [15]. It is registered under the TR-DGU project ID 2023-007 as well as at ClinicalTrials.gov (protocol ID: NCT06464289). The investigation was conducted in accordance with local ethics and data protection guidelines, with the approval given by the ethics board Westfalen-Lippe (2024-006-f-S) and in accordance with the Declaration of Helsinki and its amendments.

## Results

### Case identification and baseline characteristics

**Fig. 1:**
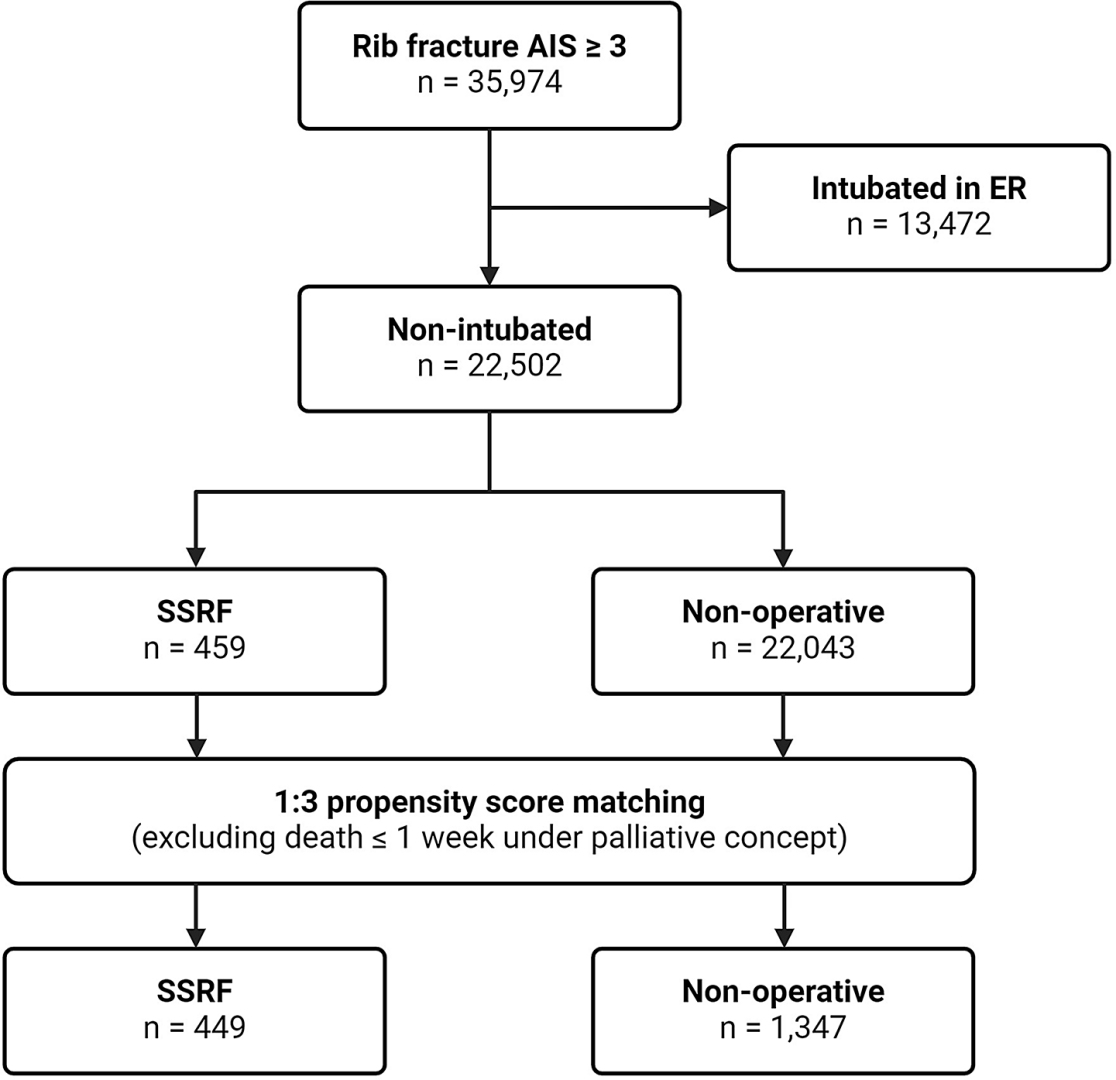
Flow diagram of case identification and matching process. AIS abbreviated injury scale, ER emergency room, SSRF surgical stabilization of rib fractures

The search of the TR-DGU yielded 35,974 patients with a rib fracture AIS ≥ 3 between 2013 and 2022. Of these, 13,372 were intubated upon arrival to the hospital or in the emergency room. Of the non-intubated patients, 459 received SSRF and 22,043 were treated conservatively. A flowchart of the case selection process is shown in Fig. 1.

The baseline characteristics, demographics and injury patterns of the SSRF and the conservative cohorts are shown in Table 1. The largest differences between the cohorts pre-matching were the presence of haemothorax (53 vs. 18%), pneumothorax (64 vs. 40%), scapula fracture (25 vs. 14%) and lung laceration (10 vs. 2%). The rate of patients classifying as a severe trauma (ISS ≥ 16) and the mean ISS were significantly higher in the SSRF cohort (82% vs. 64%; *p* < 0.001 and 22.7 vs. 20.1; p = < 0.001).

**Table 1:** Characteristics of the patient collective pre- and poste propensity matching given as number of events (n) with percentage of the total (%) or mean with standard deviation. Significance was calculated using chi-squared test or Mann-Whitney U-test; *p* < 0.05 was defined as statistically significant. SSRF surgical stabilisation of rib fractures, ASA American society of anaesthesiologists physical status classification, AIS abbreviated injury scale, SD standard deviation, ISS injury severity score, RISC II revised injury severity classification II, ER emergency room

		All patients		1:3 propensity matched cohorts			
		SSRF (*n* = 459)	Conservative (*n* = 22043)	SSRF (*n* = 449)		Conservative (*n* = 1347)	*p*-value
**Demographics** [n (%)]							
Age group							
	1–15	1 (< 1)	118 (< 1)	1 (< 1)		4 (< 1)	0,61
	16–59	206 (45)	11,480 (52)	205 (46)		581 (43)	
	60–69	124 (27)	4106 (19)	119 (27)		313 (23)	
	70–79	78 (17)	3387 (15)	75 (17)		263 (20)	
	≥ 80	50 (11)	2935 (13)	49 (11)		186 (14)	
Male gender		361 (79)	16,436 (75)		449 (79)	1051 (78)	0.84
ASA 3 or 4		95 (21)	3867 (18)		92 (21)	268 (20)	0.79
Trauma centre							
	Level 1	376 (82)	17,313 (79)	369 (82)		1208 (90)	**< 0.001**
	Level 2	71 (16)	3697 (17)	69 (15)		110 (8)	
	Level 3	12 (3)	1033 (5)	11 (2)		29 (2)	
**Thorax injuries** [n (%)]							
Rib fracture							
	AIS = 3	198 (43)	18,877 (86)	198 (44)		520 (39)	0.103
	AIS = 4	198 (43)	2111 (10)	189 (42)		608 (45)	
	AIS = 5	63 (14)	1055 (5)	62 (14)		219 (16)	
Haemothorax		245 (53)	3950 (18)		235 (52)	650 (48)	0.141
Pneumothorax		292 (64)	8900 (40)		283 (63)	888 (66)	0.28
Sternum fracture		64 (14)	2663 (12)		64 (14)	176 (13)	0.52
Clavicle fracture		115 (25)	4189 (19)		111 (25)	298 (22)	0.27
Scapula fracture		113 (25)	3168 (14)		110 (25)	272 (20)	0.062
Lung laceration		47 (10)	378 (2)		37 (8)	98 (7)	0.54
Lung contusion							
	none	283 (62)	15,055 (68)	274 (61)		858 (64)	0.37
	AIS 2	64 (14)	3325 (15)	64 (14)		209 (16)	
	AIS 3	87 (19)	3097 (14)	86 (19)		215 (16)	
	AIS 4	25 (5)	566 (3)	25 (6)		65 (5)	
**Other injuries** [n (%)]							
AIS head ≥ 3		100 (22)	6290 (29)		99 (22)	156 (12)	**< 0.001**
AIS abdomen ≥3		76 (17)	3801 (17)		76 (17)	285 (21)	0.57
AIS pelvis ≥3		66 (14)	3658 (17)		64 (14)	241 (18)	0.082
AIS spine ≥3		157 (34)	8353 (38)		153 (34)	552 (41)	**0.009**
AIS leg ≥3		70 (15)	3836 (17)		69 (15)	213 (16)	0.88
AIS arm ≥ 3		234 (51)	9394 (42)		228 (51)	625 (46)	0.107
ISS ≥ 16		375 (82)	14,222 (65)		365 (81)	1101 (82)	0.83
**Treatment pre-hospital and in the ED** [**n (%)]**							
Thoracic decompression							
	Prehospital	11 (3)	224 (1)	10 (2)		26 (2)	0.67
	ED	149 (33)	3464 (16)	143 (32)		449 (33)	0.56
Transfusion in ED		21 (4,6)	797 (3.6)		21 (5)	72 (5)	0.58
**Scores**							
ISS [mean (SD)]		22.7 (9.2)	20.1 (8.9)		22.7 (9.3)	23.7 (9.8)	**0.043**
Predicted intra-hospital mortality based on RISC II [mean (SD)]		4.0%	5.3%		5,2%	6,1%	0.089

### Propensity score

The factors, coefficients and odds ratios (OR) of the propensity score are shown in Table 2. The strongest predictors of SSRF were severe thoracic injury (AIS = 4 and 5; OR of 4.72 and 6.40, respectively), lung lacerations (OR 3.25) and haemothorax (OR 3.22). Relevant head injury (AIS ≥ 3) was a negative predictor (OR 0.70). The propensity score yielded predictions between 0% and 55% for patients receiving SSRF. After 1:3 matching 449 patients receiving SSRF and 1347 conservatively treated patients (total 1796) were included in the evaluation.

**Table 2:** Propensity score used for matching calculated using a logistic regression model with SSRF as dependent variable. The effect of each predictor is presented as odds ratio, with p-value. The probability to receive an operative rib stabilization (propensity score) is calculated by adding the respective coefficients and transforming this score into a probability by using the logistic function. AIS abbreviated injury scale, ER emergency room

Factor	Coefficient	Odds Ratio	*p*-value
Rib AIS 4	1.857	6.40	**< 0.001**
Rib AIS 5	1.552	4.72	**< 0.001**
Age ≥ 60 years	0.341	1.42	**< 0.001**
Head AIS ≥ 3	-0.353	0.70	**0.003**
Pneumothorax	0.334	1.40	**0.003**
Haemothorax	1.169	3.22	**< 0.001**
Lung laceration	1.179	3.25	**< 0.001**
Scapula fracture	0.323	1.381	**0.005**
Chest tube in ER	0.203	1.225	0.079
Trauma centre level 1	0.359	1.432	**0.005**
Constant	-5.567	--	

After propensity score matching the only significant differences between the SSRF and the conservative cohorts were the trauma centre level in which the patients were treated, the head and spine AIS scores, and the ISS. Both cohorts were largely treated in level 1 trauma centres, however 15% of the SSRF cohort and only 8% conservative cohort were treated in level 2 trauma centres. Head AIS ≥ 3 was more common in the SSRF cohort (22% vs. 12%; *p* < 0.001), whilst spine AIS ≥ 3 was more common in the conservative cohort (34% vs. 41%; *p* = 0.009). Finally, the ISS was marginally yet significantly lower in the SSRF than in the conservative cohort (22.7 ± 9.3 vs. 23.7 ± 9.8; *p* = 0.043). All other variables were comparable between the groups post-matching (see Table 1).

### Outcomes

The outcomes of the SSRF and the conservative cohort are shown Table 3. The SSRF cohort showed significantly decreased in-hospital mortality compared to the conservative cohort (1.6% vs. 4.8%; *p* = 0.002) and compared to the RISC II prognosis (5.2%). However, the number of days on the ventilator was significantly higher in the SSRF than in the conservative cohort (3.9 ± 9.6 vs. 2.5 ± 7.9 days; *p* < 0.001). Also, the overall duration in the ICU was higher in the SSRF cohort (9.6 ± 12 vs. 6.9 ± 9.8 days; *p* < 0.001).

**Table 3:** Outcomes of the 1:3 propensity matched cohort given as number of events (n) with percentage of the total (%) or median with first and third quartiles (Q_1_– Q_3_) as appropriate. SSRF surgical stabilisation of rib fractures, ICU intensive care unit, OF organ failure

		Matched cohorts		
		SSRF *n* = 449	Conservative *n* = 1347	*p*-value
Predicted Mortality by RISC II score [%]		5.2	6.1	
Outcome				
	Mortality [n (%)]	7 (1.6)	64 (4.8)	**0.002**
	OF, single [n (%)]	121 (31)	271 (23)	**< 0.001**
	OF, multiple [n (%)]	59 (15)	146 (12)	0.164
	OF, lung [n (%)]	73 (19)	137 (12)	**< 0.001**
	Sepsis [n (%)]	30 (7.8)	66 (5.7)	0.144
Treatment				
	Intubated on ICU [n (%)]	169 (39)	336 (27)	**< 0.001**
	Ventilation (days) [median (Q1-Q_3_)]	0 (0–2)	0 (0–0)	**< 0.001**
	Duration ICU (days) [median (Q1-Q_3_)]	5 (2–12)	3 (1–8)	**< 0.001**
	Hospital stay (days) [median (Q1-Q_3_)]	18 (13–26)	14 (9–22)	**< 0.001**
Glasgow Outcome Scale				**< 0.001**
	Persistent vegetative state [n (%)]	1 (< 1)	6 (< 1)	
	Severe disability [n (%)]	36 (8)	67 (5)	
	Moderate disability [n (%)]	121 (27)	291 (22)	
	Good recovery [n (%)]	227 (63)	1327 (68)	

With respect to the secondary outcomes, SSRF showed significantly increased rate of secondary intubation and longer hospital stay. Furthermore, they developed OF of the lung (19% vs. 12%; *p* < 0.001) and single OF (31% vs. 23%; *p* < 0.001) more frequently than the conservative cohort. There was no significant difference in the rate of multiple OF or sepsis between the cohorts. Finally, the SSRF cohort showed reduced outcomes at discharge according to the GOS, with however, comparable rate of good recovery (63% vs. 68%).

**Table 4:** Subgroup analysis of mortality, rate of intubation, intensive care unit and hospital length of stay (ILOS and HLOS) as well as the rate of respiratory failure with respect to age, the injury severity score (ISS) and the pre-injury American Society of Anaesthesiologist physical status classification (ASA) score. The data is given as number of patients and total number of patients with percentage or as median with quartiles (Q_1_– Q_3_). * *n* = 2; only median reported

		Mortality [n / total (%)]		Intubated on ICU [n (%)]		ICU LOS [median (Q_1_– Q_3_)]		HLOS [median (Q_1_– Q_3_)]		Respiratory failure [n / total (%)]	
		SSRF	Cons.	SSRF	Cons.	SSRF	Cons.	SSRF	Cons.	SSRF	Cons.
Age [years]	< 60	2/206 (1.0)	6/585 (1.0)	66/193 (34)	134/538 (25)	5 (2-10)	3 (1-7)	16 (12-24)	13 (9-20)	28/175 (16)	42/512 (8)
	60 - 85	4/234 (1.7)	41/694 (5.9)	100/230 (44)	185/639 (29)	6 (2-14)	4 (2-9)	20 (14-29)	15 (10-23)	42/204 (21)	86/616 (14)
	> 85	1/9 (11.1)	17/68 (25.0)	3/9 (33)	17/59 (29)	9 (4-14)	4 (2-11)	14 (10-32)	13 (7-23)	3/8 (38)	9/55 (16)
ISS	< 16	1/84 (1.2)	6/246 (2.4)	8/80 (10)	24/220 (11)	3 (2-7)	2 (1-5)	15 (11-21)	11 (7-17)	2/71 (3)	11/214 (5)
	16 - 24	2/206 (1.0)	19/583 (3.3)	63/195 (32)	97/518 (19)	4 (2-9)	3 (1-6)	16 (13-25)	12 (9-19)	28/172 (16)	39/498 (8)
	≥ 25	2/206 (1.0)	19/583 (3.3)	98/157 (62)	215/498 (43)	10 (4-17)	6 (3-13)	22 (16-32)	19 (13-28)	43/144 (30)	87/471 (19)
Pre-injury ASA	1	4/159 (2.5)	39/518 (7.5)	54/146 (37)	109/450 (24)	5 (2-10)	3 (1-7)	16 (12-24)	13 (9-20)	20/132 (15)	35/434 (8)
	2	1/157 (0.6)	4/493 (0.8)	66/162 (41)	98/409 (24)	5 (2-12)	3 (2-7)	17 (13-26)	15 (10-22)	33/143 (23)	42/398 (11)
	3	4/90 (4.3)	32/261 (12.3)	35/88 (40)	91/248 (37)	9 (3-18)	6 (2-12)	22 (14-39)	15 (9-24)	16/81 (20)	45/240 (19)
	4	1/2 (50)	53/172 (30.8)	1/2 (50)	1/7 (14)	26.5*	3 (2-5)	31.5*	11 (6-22)	1/2 (50)	1/6 (17)

### Subgroup analysis

Exploratory subgroup analysis with respect to age, ISS and pre-injury American Society of Anaesthesiologists physical status classification (ASA) was performed (Table 4). With respect to mortality, the subgroup analysis showed higher mortality in older patients irrespective of treatment. Simultaneously, the largest difference in mortality between the SSRF and the conservative cohorts was observed in the > 85 age group, with 13.9% lower mortality after SSRF. The mortality rate showed little difference between the two groups with respect to the ISS, with only slightly lower mortality after SSRF. At ISS ≥ 25 this effect was the largest (2.5% vs. 7.5%). ASA 1 patients showed low mortality rates in both groups (0.6% vs. 0.8%). For ASA 2 and 3 patients the mortality rate was lower after SSRF than after conservative treatment (0% vs. 4.3% and 3.6% vs. 12.3% respectively). The mortality of ASA 4 patients was high in both groups.

The rate of secondary intubation, ILOS and HLOS were generally higher in the SSRF than in the conservative cohort for all age, ISS and ASA groups. There was a slight trend towards a larger difference in the older age groups and at higher ISS and ASA scores. The rate of respiratory failure was also generally higher in the SSRF than in the conservative cohorts with a more pronounced effect at higher age, and with higher ISS and ASA scores.

**Fig. 2:**
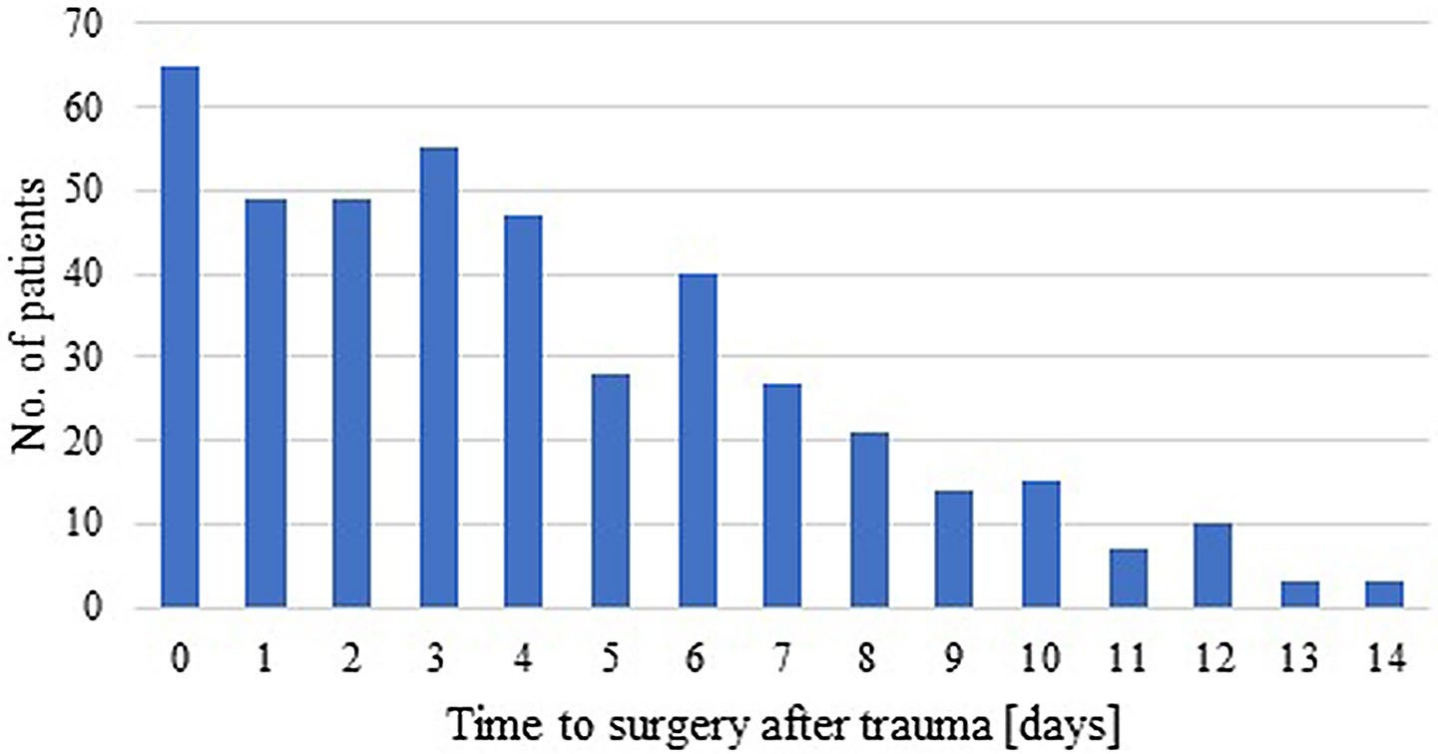
Analysis of time point to surgery. The number of patients with surgery on a particular day is shown from the day of trauma (day 0) up to 14 days after trauma

An analysis of time to surgery is shown in Fig. 2. This showed a range of the time point of SSRF between 0 and 35 days after injury. 25% of surgeries were performed on the day of admission or on the day thereafter. In order to differentiate between patients that developed different forms of organ failure and those who did not within the SSRF cohort, these subgroups were compared directly with respect to their time to surgery (Table 5). The mean time to surgery in SSRF patients that did not develop organ failure was 5.0 ± 4.4 days and it consistently increased for patients that developed any organ failure (5.5 ± 5.0 days), lung failure (6.0 ± 6.5 days), MOF (6.7 ± 6.9 days) and MOF with lung failure (6.8 ± 7.3 days). This increase in the time to surgery was accompanied by a decrease in the number of patients with surgery on the day of injury or on the day thereafter.

**Table 5:** Analysis of the timepoint of surgery in the subgroup of patients with different types of organ failure (OF) and multiple organ failure (MOF). Given is the mean time to surgery with the standard deviation (SD) and the number as well as percentage of patients with early surgery (day 0 or 1)

	Number of patients	Mean time to surgery [days (SD)]	Patients with early surgery [*n* (%)]
No OF	273	5.0 (4.4)	61 (22)
Any OF	123	5.5 (5.9)	28 (23)
Lung failure	75	6.0 (6.5)	14 (19)
MOF	61	6.7 (6.9)	9 (15)
MOF with lung failure	51	6.8 (7.3)	9 (18)

## Discussion

Here data from 22,502 patients with severe rib trauma (AIS ≥ 3) not intubated on admission or in the ED was evaluated. Of these, 459 were treated with SSRF, which to our knowledge is the largest published dataset of non-intubated patients to undergo SSRF [12–14]. This corresponds to a SSRF rate of 2%, which is slightly higher than the 1.6% reported by Becker et al. from a partly overlapping dataset of the TR-DGU (2008 to 2017) considering intubated and non-intubated patients, showing a trend towards more SSRF [16]. It is slightly lower than the SSRF rate of 4.5% in flail chest patients reported by a retrospective Canadian study [17].

Our investigation showed a lower mortality in the SSRF group, which is in agreement with current systematic reviews of all studies investigating SSRF [10, 11], but has thus far not been demonstrated in non-intubated patients [12–14]. This decreased mortality is also pronounced when compared to the predicted in-hospital mortality given by the RISC II score (actual mortality 1.6% versus predicted mortality 5.2%) [18].

The mean ISS in the SSRF cohort was 22.7 and no restrictions were placed on accompanying injuries. In contrast, Pieracci et al. in their study of SSRF in non-ventilator dependent patients excluded patients with flail chest, severe pulmonary contusion and pelvic fractures expected to require surgery, leading to a lower mean ISS of 13 in the operative group [12]. Similarly, the median ISS in the studies by Marasco et al. and Ali-Osman et al. were 14 and 17.5 respectively [13, 14]. This could explain why these previous studies of non-intubated patients did not show a survival benefit. This is in line with the currently available evidence showing the greatest benefit of SSRF in the most severely injured patients and especially in those patients flail chest deformities [10].

Surprising, despite the decreased mortality in the SSRF cohort, this group showed an increased rate of OF particularly of the lung as well as increased ILOS and HLOS. This is an effect that has previously been described in another large retrospective dataset [17], but is generally in contradiction to the prevailing literature opinion [10, 11]. Dehghan et al. argued that this effect may be due to selection bias leading to more severely injured patients being chosen for surgery. Furthermore, they argued that SSRF may have been performed specifically in patients failing conservative treatment, however, they could not provide data supporting these hypotheses [17].

In an attempt to elucidate the reason for increased OF, ILOS, HLOS and ventilator time, we performed subgroup analyses for potential confounders such as age, ISS and pre-injury ASA. As expected, higher age, higher ISS and higher pre-injury ASA were associated with worse outcomes. This was the case for both the SSRF and the conservative cohorts. Due to the small size of the individual subgroups this data is not conclusive, but goes some way to show that age, ISS and ASA did not cause the discrepancy between the observed mortality and complication rates.

Analysis of the complication rate in the SSRF cohort with respect to the time point of SSRF surgery showed a tendency towards longer mean time to SSRF and lower rates of early SSRF in the subgroups with more severe OF. This is in line with the current evidence, which shows better results after early SSRF [10, 19, 20]. Due to the retrospective nature of this investigation no clear conclusion can be drawn with respect to the reason for the late time point of SSRF. It is possible that the late timepoint of surgery is the cause for the increased rate of OF observed. On the other hand, it is also possible that failure of conservative management with beginning or manifest OF was a reason for a crossover into the SSRF cohort, as has previously been argued [17].

Generally, the late timepoint of SSRF observed here stands in contradiction to the existing recommendations, but has been previously shown for an older TR-DGU dataset [16, 21]. It might be expected that the rate of organ failure would decrease in non-intubated patients in response to earlier SSRF [20]. This hypothesis is supported by an analysis of the TR-DGU showing that earlier SSRF within 48 h after trauma is associated with reduced ILOS and HLOS as well as a trend towards shorter duration of mechanical ventilation in comparison to late SSRF between 3 and 10 days after trauma [22].

## Strengths and limitations

The nature of the dataset used here leads to both strengths and limitations. The retrospective nature and the large number of participating centres in the dataset makes it representative of the treatment reality in the regions participating in the TR-DGU in a way that a prospective study limited to a small number of study centres would not. This leads to good generalisability and external validity of the data. It also leads to a large sample size, thus enabling the investigation of more different factors.

However, the dataset provided by the TR-DGU was not conceived to specifically reflect the degree of thoracic injury, such as the precise morphology of the rib fractures. Furthermore, the dataset does not give insight into the reasoning behind therapy decisions and timing. For example, the degree of clinical instability or a potential lack of resources in the primary hospital admitting the patients are not reflected in the dataset. Therefore, it remains unclear, if the late timepoint of surgery is due to the patients being too unstable for earlier surgery or due to the patients failing initial conservative management. This question requires studies to be more clearly elucidated. Furthermore, the surgical technique and surgical approach used for SSRF could not be analysed based on the available data and other thoracic surgical procedures concomitant with SSRF were not assessed, all of which may have an impact on the outcome parameters. The set of available outcomes is also limited, with no data available on functional pulmonary parameters or pain.

Generally, the dataset is suitable to describe the treatment reality and the outcomes after SSRF. However, more in-depth analyses regarding possible causality and subgroup effects must remain exploratory in nature.

## Conclusion

To our knowledge this is the first investigation to show decreased mortality due to SSRF in non-intubated patients. The data supports that more severely injured patients profit most from SSRF. Furthermore, it supports that early SSRF should be preferred over late SSRF. Finally, the data indicates that SSRF may also be a useful strategy in patients that have failed initial conservative management, however, this must remain a conjecture based on the available data. Further detailed investigations are required to elucidate which non-intubated patients may benefit most from SSRF and what effect the surgical technique and surgical timing have on the outcomes. This could for example be realised in a prospective multi-centre study comparing surgical techniques and timepoints.

## Data Availability

No datasets were generated or analysed during the current study.
